# Revision of endemic Marquesas Islands *Bidens* (Asteraceae, Coreopsideae)

**DOI:** 10.3897/phytokeys.38.7609

**Published:** 2014-06-04

**Authors:** Warren L. Wagner, John R. Clark, David H. Lorence

**Affiliations:** 1 Department of Botany, MRC-166, National Museum of Natural History, Smithsonian Institution, P.O. Box 37012, Washington, DC 20013-7012, USA; 2 National Tropical Botanical Garden, 3530 Papalina Road, Kalaheo, HI 96741-9599, USA

**Keywords:** *Bidens*, Asteraceae, Coreopsideae, Marquesas Islands, French Polynesia, conservation

## Abstract

During the preparation of the *Vascular Flora of the Marquesas Islands* four new species of *Bidens* (Coreopsideae, Asteraceae) have come to light and are described herein: *Bidens woodii* W.L. Wagner, J.R. Clark & Lorence, **sp. nov.** from Ua Pou, and *Bidens microcephala* W.L. Wagner, J.R. Clark & Lorence **sp. nov.**, *Bidens evapelliana* W.L. Wagner, J.R. Clark & Lorence, **sp. nov.,** and *Bidens wichmanii* W.L. Wagner, J.R. Clark & Lorence, **sp. nov.** from the undercollected island of Fatu Hiva. In addition to these new species, we recognize the following six species of *Bidens* previously described from the Marquesas Islands: *Bidens beckiana* (F. Br.) Sherff (Eiao and Hatutaa), *Bidens bipontina* Sherff and *Bidens cordifolia* Sch. Bip. (both in Nuku Hiva), *Bidens henryi* Sherff (Hiva Oa, Tahuata), *Bidens uapensis* (F. Br.) Sherff (Ua Pou), and *Bidens polycephala* Sch. Bip. (Nuku Hiva, Ua Huku, Hiva Oa, Tahuata, and Mohotani). Two names are reduced to synonymy under *Bidens polycephala*: *Bidens collina* Degener & Sherff, syn. nov. and *Bidens jardinii* Sch. Bip., syn. nov. *Bidens polycephala* has the widest distribution of the Marquesan species on five of the islands and exhibits considerable variation.

## Introduction

The Flora of the Marquesas Islands project is a collaborative program, primarily between the Smithsonian Institution and the National Tropical Botanical Garden, intended to further knowledge of the flora of this remote archipelago. In 1997 the first publications of new species and revisions of genera with more than one endemic species were initiated (Florence and Lorence 1997; Wagner and Lorence 1997). Since that time a series of publications has enumerated and revised a number of genera (for summary see Lorence and Wagner 2011). After the 2011 issue in PhytoKeys there were only two major groups in the Marquesas Islands flora for which revisions had not been completed. The first, *Cyrtandra* J.R. Forst. & G.Forst. (Gesneriaceae), is now complete with 11 species (Wagner et al. 2013). The present revision of the Marquesas species of *Bidens* L. completes the precursor publications required to finalize the data in the online Flora of the Marquesas Islands website (Wagner and Lorence 2002 and onward; http://botany.si.edu/pacificislandbiodiversity/marquesasflora/index.htm ). The new species described in these precursor publications have increased the known native flora in the Marquesas Islands by about 25%.

The genus *Bidens* is the largest genus of the tribe Coreopsideae with 150–235 species (Sherff 1937; Strother and Weedon 2006); most research on various groups within the genus suggest numbers at the lower end of the range are probably more accurate (e. g. Ganders and Nagata 1990). The genus has had a long, complex taxonomic history summarized by Sherff (1937) in his revision of the genus. Sherff’s revision remains the only modern study of the entire genus as presently delimited in which he treated 235 species, many with one to several varieties. Most of the species occur in the Americas and Africa, while Sherff (1937) accepted 59 species in the Hawaiian Islands and southeastern Polynesia, but our current estimate is there are about 40 species. There are only a few indigenous species in Europe and northern Asia. The taxonomy of the genus remains confused despite a growing number of individual groups within the genus having been the subject of detailed studies (Ganders et al. 2000). Species of related genera such as *Coreocarpus* Benth., *Coreopsis* L., *Cosmos* Cav., *Dahlia* Cav., and *Thelesperma* Less. have had a tangled history with *Bidens*, and it has been one of the most difficult to define. Relationships among the 20+ genera within the tribe Coreopsideae are poorly understood, but recent phylogenetic studies using molecular data as well as morphological and anatomical traits are helping to resolve monophyletic groups within the tribe and the relationships among them (Ganders et al. 2000; Kimball and Crawford 2004; Crawford et al. 2009; Knope et al. 2012; Funk et al. unpubl.). These recent molecular phylogenies have also indicated that some Coreopsideae genera are not monophyletic, especially in the broadly sampled (multiple species in 19 genera) study by Kimball and Crawford (2004). These studies have resolved a number of well-supported lineages; however, the two largest genera, *Bidens* and *Coreopsis*, were determined to be non-monophyletic in their present delimitation. One alternative at this stage of the phylogenetic understanding of the various clades is that a broadly circumscribed genus *Bidens* (or *Coreopsis*), inclusive of much of the tribe, would be the best taxonomic solution to the problem. The alternative would be to segregate out numerous relatively small genera reflecting morphologically identifiable monophyletic groups. If this latter course were to be followed for the Pacific species, which are in a clade containing *Bidens pilosa* L. and other New World species, the correct generic name would seem to be the obscure name *Kerneria* Moench.

**Figure 1.**
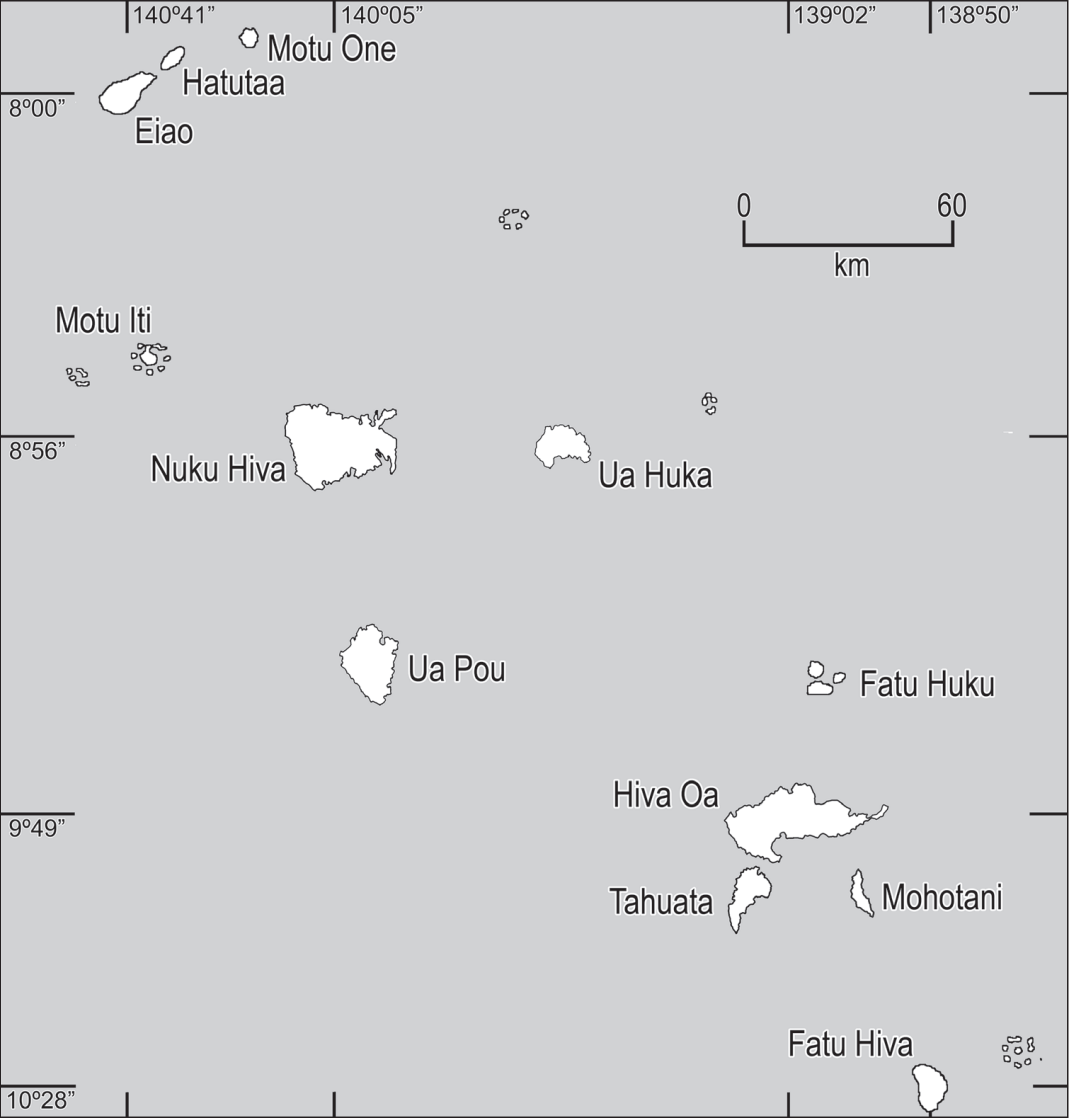
Map of the Marquesas Islands.

In the Marquesas Islands Schultz (1856) described four species, *Bidens cordifolia*, *Bidens jardinii*, *Bidens polycephala*, and *Bidens serrulata*, in his enumeration of plants collected by Jardin. *Bidens jardinii* is here considered to be a synonym of *Bidens polycephala*, and *Bidens serrulata* is a homonym. Sherff as part of his overall multiyear efforts to study the genus provided *Bidens serrulata* with the new name, *Bidens bipontina*. Forest Brown (1935) described two additional species in the genus *Campylotheca* Cassini, *Campylotheca beckiana* and *Campylotheca uapensis*. These were transferred later to *Bidens* by Sherff, who also described three additional species himself, *Bidens henryi*, *Bidens collina*, and *Bidens ahnnei* (the latter two here considered synonyms of *Bidens polycephala*). Additional names in *Bidens* from the Marquesas are *Bidens hivoana* Degener & Sherff (1934), described from the island of Hiva Oa, and *Bidens teikiteetinii* Florence and Stuessy (1988). With subsequent study both have been transferred to *Oparanthus* (Shannon and Wagner 1997). To the previously described six species we here add four species from two underexplored islands: *Bidens woodii*, from the summit of Ua Pou, and *Bidens microcephala*, *Bidens evapelliana*, and *Bidens wichmanii* from Fatu Hiva (Table 1). We have arranged the species in the taxonomic section according to an unpublished phylogeny by Funk et al. See Fig. 1 for map of the Marquesas Islands showing the geographic relationships among the islands.

**Table 1.** Island distribution of Marquesas species of *Bidens*. Islands arranged from oldest to youngest.

Species / Island	Eiao	Hatutaa	Nuku Hiva	Ua Huka	Ua Pou	Hiva Oa	Tahuata	Fatu Hiva
*Bidens henryi*						X	X	
*Bidens polycephala*			X	X		X	X	
*Bidens uapensis*					X			
*Bidens microcephala*								X
*Bidens woodii*					X			
*Bidens evapelliana*								X
*Bidens wichmanii*								X
*Bidens bipontina*			X					
*Bidens cordifolia*			X					
*Bidens beckiana*	X	X						
**Totals**	1	1	3	1	2	1	2	3

When evaluated using the IUCN criteria for endangerment (IUCN 2001, see also www.iucnredlist.org/info/categories_criteria2001 ), most of the Marquesan species of *Bidens* fall into the Endangered (EN) or Critically Endangered (CR) categories, which designate species facing the highest risk of extinction in the wild. Marquesan species of *Bidens* meet these IUCN criteria by having known ranges less than 100 km^2^, an area of occupancy of less than 10 km^2^, and continuing decline in the quality of habitat across the Marquesas Islands.

Due to the small areas involved on oceanic islands, many single island endemics have a geographic range of less than 100 km² and some less than 10 km². Therefore they will fall automatically into the Critically Endangered (CR), Endangered (EN), or Vulnerable (VU) category, or in other words into extremely high (CR) to lower (VU) risk of extinction categories despite the fact that they may have relatively small natural ranges and may be relatively common locally with healthy, regenerating populations. Some will fall into the Data Deficient (DD) category, simply because we know so little about their actual population size, status, and range. Although the IUCN evaluations are not ideally suited for islands, for the sake of consistency we use them herein for Marquesas *Bidens* and where appropriate have added qualifying comments under Conservation Status for species that seem extremely at risk and those that are more common.

All measurements given herein are taken from dried herbarium specimens, although certain features such as shapes were supplemented with information from alcohol-preserved flowers and fruits, field notes, and color slides or digital photos. Measurements are presented in the descriptions as follows: length × width, followed by units of measurement (mm or cm). Specimens from the following herbaria were studied: AD, BISH, BKL, BR, CBG, CHR, F, K, L, MO, MPU, NSW, NY, P, PAP, PTBG, OS, TEX, UBC, US, and WU. We have examined all collections cited except for the four types of the Schultz (1856) names for which we studied images of them sent by P. Lowrey.

## Taxonomic part

### Artificial key to species of Marquesas *Bidens*

**Table:** 

1	Ray florets absent; disk florets 1–10; heads < 2 mm in diameter	4. *Bidens microcephala*
–	Ray florets present; disk florets 12–50; heads > 2 mm in diameter	2
2	Plant prostrate; leaf blades 1–2.8(–5.5) cm	6. *Bidens evapelliana*
–	Plant ascending to erect; leaf blades (1–)3–12 cm	3
3	Leaves thick and coriaceous, veins raised on lower surface, blades ovate	5. *Bidens woodii*
–	Leaves membranous to chartaceous, veins not conspicuously raised, blades lanceolate to elliptic, rarely narrowly ovate	4
4	Leaf blades with a tuft of hairs at base on lower surface	3. *Bidens uapensis*
–	Leaf blades glabrous or sometimes pubescent, but not with hairs in a tuft	5
5	Heads 8–15 mm in diameter; disk florets ca. 50	7. *Bidens wichmanii*
–	Heads 2–15 mm in diameter; disk florets 12–34	6
6	Outer involucral bracts 1–4.5 mm long; disk florets 12–25; rays 3–12 mm long	2. *Bidens polycephala*
–	Outer involucral bracts (4–)5–13 mm long; disk florets 30–34; rays 9–20 mm long	7
7	Heads 2–5 mm in diameter; rays 13–20 mm long; leaf apex long-acuminate to caudate	8. *Bidens bipontina*
–	Heads 5–15 mm in diameter; rays 9–15 mm long; leaf apex acute to acuminate or sometimes caudate	8
8	Outer involucral bracts recurved; plant glabrous except sometimes a few hairs on the involucral bracts	.1. *Bidens henryi*
–	Outer involucral bracts erect to ascending; plant pubescent, at least along the petiole or involucral bracts, but often also on leaves	9
9	Outer involucral bracts similar to inner ones and tapering at base; leaves simple, glabrous or pubescent toward the base and in a line along the petiole	10. *Bidens beckiana*
–	Outer involucral bracts well differentiated from inner ones and pales, not tapering at the base; leaves trifoliolate or sometimes simple, moderately pubescent, especially on lower surface, midrib, veins, and in a line along the petiole	9. *Bidens cordifolia*

#### 1. Bidens henryi

Sherff, Bot. Gaz. (Crawfordsville) 76: 164. 1923.

http://species-id.net/wiki/Bidens_henryi

Fig. 2D–F

Campylotheca henryi (Sherff) F. Br., Bernice P. Bishop Mus. Bull. 130: 355. 1935.

##### Type.

Marquesas Islands. Hiva Oa: Atuona Valley ridge on route to Hanamenu, 3500–4000 ft [1065–1220 m], December 1917, C. Henry s.n. (holotype: F-474749!).

##### Description.

Erect subshrubs to shrubs 1–2 m tall. Leaves simple, 2.5–13 cm long including petiole, blades lanceolate or elliptic-lanceolate, 1.8–9.5 × 0.5–3.8 cm, glabrous, margins serrate, apex acuminate. Heads (1–)3–4(–7), in diffuse cymes terminating main stem and lateral branches, 5–15 mm in diameter excluding rays, peduncles 1.2–9.5 cm long, glabrous; outer involucral bracts 4–13 mm long, linear, well differentiated from inner bracts, recurved, apex erose, sometimes with few short hairs; ray florets ca. 13, sterile, rays yellow, 12–15 × 2–5 mm; disk florets ca. 30–32, perfect, corollas yellow. Achenes black, straight, 8–10 × 1–2 mm, glabrous or with few inconspicuous setae near the apex; pappus of 2–3 antrorse barbed awns.

**Figure 2.**
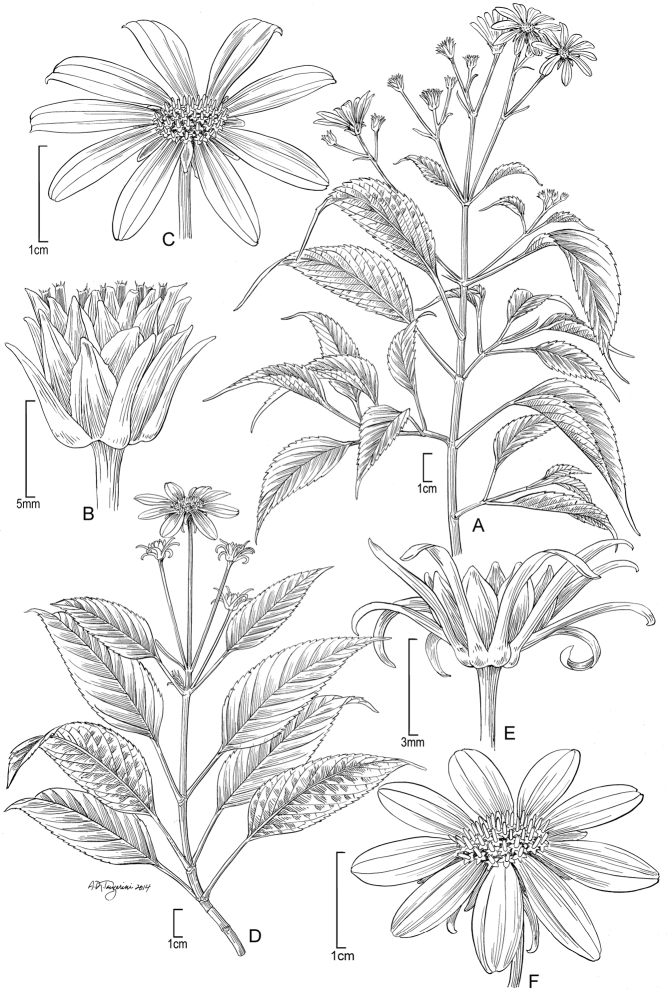
*Bidens bipontina* Sherff (**A–C**). **A** Distal part of plant **B** Head post-flowering showing involucral bracts and apex of achenes **C** Head showing rays and apex of disk florets. Drawn from Perlman & Wood 15028 (US), except B from Wood and Perlman 4601 (US). Images used to augment illustration from field shots of Wood and Perlman 4601 and Perlman and Wood 15021. *Bidens henryi* Sherff (**D–F**). **D** Distal part of plant **E** Head post-flowering showing involucral bracts **F** Head showing rays and disk florets. Drawn Wood 10038 (US), except F from Perlman 14868 (US). Images from field shots used to augment illustration of Wood 10038, Wagner 6219 and Lorence & Price 8932.

##### Distribution.

Marquesas Islands, occurring on Hiva Oa and Tahuata, (400-) 620–1200 m.

##### Habitat.

*Bidens henryi* is known from ridges and summit areas in montane wet shrubland or low forest with *Metrosideros collina* (J.R. Forst. & G. Forst.) A. Gray and *Weinmannia marquesana* F. Br. with fern understory along with other shrubs and trees such as species of *Cheirodendron*, *Coprosma*, *Crossostylis*, *Cyrtandra*, and *Myrsine*.

**C**

##### onservation status.

Proposed IUCN Red List Category **Endangered** (EN), criteria B2a, B2b (i–iii): B2: total area of occupancy less than 500 km² (ca. 378 km²); B1a, severely fragmented; B1b (i–iii), habitat continuing decline inferred. The suitable habitat for *Bidens henryi* on Hiva Oa (ca. 315 km²), and Tahuata (ca. 61 km²) is restricted to mountain slopes and summits, indicated as an endangered environment that is threatened by human activity (deforestation and fire), feral animals, and invasive plants, thus reducing the extent of the forest. It is relatively common locally in some areas, however, and consequently vulnerable (VU) might be an alternative category (Butaud, pers. comm. 2014).

##### Specimens examined.

**Marquesas Islands. Hiva Oa:** Chemin d’Atuona à Hanamenu par Feani, crête Hanamenu, 1090 m, 12 Feb 1975, Schäfer 5192 (K, US); Chemin d’Atuona à Hanamenu par Feani, côté Atuona, 1010 m, 13 Feb 1975, Schäfer 5205 (K, MPU); Feani ridge to upper slopes of dry side of island, 1150 m, 12 Feb 1975, Oliver & Schäfer 3122 (BISH, MO, P, PTBG, US); Feani, trail from Atuona to Hanamenu, 1190 m, 10 Feb 1975, Oliver & Schäfer 3105 (BISH, P, PTBG, US); Feani, vieux sentier d’Atuona à Hanamenu, crête côté Hanamenu, 900 m, 5 Mar 1975, Schäfer & Oliver 5271 (K, MPU, US); Puamau, chemin vers Atuona, après la bifurcation vers Hanaupe, lieu-dit Keiani, 650 m, 26 Mar 1975, Schäfer 5382 (K, MPU, US); A l’est de la piste d’aviation, 7 Mar 1974, Hallé 2117 (US); Mt. Ootua, central part, 785 m, 28 Jul 1977, Gagné 1200 (BISH, US); Mt. Ootua, central part, 800 m, 27 Jul 1977, Gagné 1166 (BISH, US); Rim of Puamau valley along trail from main road, 650 m, 9°46"26.8'S/138°54"24.8'W, 20 Feb 2003, Perlman 18488 (BISH, P, PAP, PTBG, US); NE slopes Mt. Temetiu, 700 m, 23 Feb 1929, Mumford & Adamson 49 (BISH); Atuona V., 700 m, 6 Oct 1930, Pacific Ent. Survey EX 49 (BISH [2]); Feani, 900 m, 15 Dec 1921, Brown 1084 (BISH); Road from Atuona to Puamau, just below Mt. Ootua, 700 m, 22 Jan 1975, Sachet, Oliver & Schäfer 2131 (BISH, PTBG, US); Ootua, 800 m, 15 Dec 1921, Brown & Brown 1018 (BISH); Atuona-Feani Trail, upper part of trail, exposed slope, 900–1000 m, 24-26 Sep 1963, Sachet & Decker 1122 (BISH, NSW, MO, P, US); Teavaimataii, N side Mt. Ootua, 800 m, 6 May 1929, Mumford & Adamson 351 (BISH [2]); Hanaiapa, 800 m, 30 Oct 1922, Quayle 1600 (BISH); Road from Atuona to Puamau, just below Mt. Ootua, 660–690 m, 22 Jan 1975, Sachet, Oliver & Schäfer 2129 (BISH, CBG, CHR, K, MO, NSW, L, P, PTBG, US); Entre Hanamenu et la crête de Feani, lieu-dit Teho’o-ho’o, 750 800 m, 27 Jul 1975, Schäfer 5629 (MPU); Hava Iafa, 700 m, 29 Oct 1922, Jones 1600 (BKL); Atuona-Feani Trail, upper part of trail, just below crest of ridge, 1200 m, 24–26 Sep 1963, Sachet & Decker 1141 (BISH, PTBG, US); Where trail toward Hanamenu turns into dry ridge, 884 m, 3 Aug 1988, Perlman 10208 (BISH, F, MO, PTBG, UBC, US); Trail to Feani and Hanamenu, 1006 m, 29 Jul 1988, Perlman, Wagner, Lorence, Florence & Montgomery 10177 (AD, BISH, F, MO, P, PAP, PTBG, UBC, US); Along old Atuona-Hanamenu Trail, on high ridge leading to Mt. Feani, 1050–1150 m, 30 Jul 1988, Lorence, Wagner, Florence, Perlman & Montgomery 6259 (PAP, PTBG); Mt. Feani, 1100 m, 11 Nov 1989, McKee 44683 (BISH, PAP); Atuona, piste de Hanamenu, 680 m, 29 Jul 1988, Florence, Lorence, Perlman & Wagner 9609 (BISH, P, PAP, US); trail from Atuona (W) to ridge leading to Mt. Feani and Temetiu, 1020 m, 29 Jul 1988, Wagner & Lorence 6219 (BISH, PTBG, US); Atuona, piste de Hanamenu, crête centrale, 1010 m, 29 Jul 1988, Florence, Lorence, Perlman & Wagner 9617 (BISH, P, PAP, US); Base camp, near Vaitumete to ridge crest south of Teakatau, 1200 m, 9°48"S/139°4"W, 29 Jan 2003, Lorence, Dunn & Price 8932 (BISH, P, PAP, PTBG, US)[used in molecular study, Funk et al.], cult from Lorence et al. 8932, Lorence 9175 (BISH, PTBG, US); Temetiu region, drainages to southeast of Vaimete et Vaiumioi (source), headwaters of Hanamenu, 1067 m, 29 Jan 2003, Wood 10038 (BISH, NY, P, PAP, PTBG, US); Trail to Hanamenu, along summit crest, 1000 m, 9°47"9.29'S/139°4"56.7"W, 1 Aug 2005, Perlman 19759 (PAP, PTBG); Road from Atuona to Puamau, just below Mt. Ootua, spur ridge above road below Ootua peak, 720 m, 23 Nov 1974, Sachet & Decker 1923 (BISH, CBG, CHR, K, MO, MPU, NSW, L, P, PAP, PTBG, US); Eiaone-Piamau divide, crest approx. 0.5 km from pass, 400 m, 5 Dec 1963, Decker 973 (BISH, MO, NSW, PTBG, US); Atuona-Feani Trail, Feani ridge, 1000 m, 24–26 Sep 1963, Sachet & Decker 1195 (US); Mt. Ootua, off road between Airport and Puamau, on E side of summit, 811 m, 21 Aug 1995, Perlman & Wood 14868 (BISH, MO, P, PAP, PTBG, UBC, US); Eiaone–Piamau divide, crest approx. 0.5 km from pass, 400 m, 5 Dec 1963, Decker 973 (BISH, MO, NSW, PTBG, US).**Tahuata:** Vaitahu, crête d’Amatea, début de la montée raide vers la partie haute, 620 m, 10 Apr 1975, Schäfer 5519 (MPU); Ridge between Amatea & Haaoiputeomo, summit crest of island, 823 m, 12 Jul 1997, Perlman, Wood & Luce 15989 (MO, P, PAP, PTBG, US); Summit of ridge above Vaitahu, near Haaoiputeomo, on ridge near antenna, along ridge crest between Vaitahu & Hanatetena, 823 m, 1 Sep 1995, Perlman, Wood & Luce 14917 (MO, P, PAP, PTBG, US); Along top of ridge from Amatea to Haaoiputeomo, over Hanatetena village, Summit crest of island above Vaitahu, 866 m, 11 Jul 1997, Perlman, Wood & Luce 15958 (P, PAP, PTBG, US); Région du sommet de Tahuata, 17 Mar 1974, Hallé 2186 (US); de Hamatea à la crête centrale de l’île, 750–850 m, 26 May 1975, Thibault 54 (BISH, US); Mt. Ootua, summit area and along trail from road cut, 732 m, 9°46"10'S/138°58"31.4'W, 19 Jul 2004, Perlman & Wood 19207 (BISH, P, PAP, PTBG, US).

##### Discussion.

*Bidens henryi* has conspicuous linear, recurved outer involucral bracts.

#### 2. Bidens polycephala

Sch. Bip., Flora 39: 360. 1856.

http://species-id.net/wiki/Bidens_polycephala

Figure 3E–F

Coreopsis polycephala (Sch. Bip.) Drake, Ill. Fl. Ins. Mar. Pac. 209. 1890. **Type.** Marquesas Islands. Nuku Hiva, D.E.S.A. Jardin 40 (holotype: P).
Bidens jardinii Sch. Bip., Flora 39: 360. 1856.
Coreopsis jardinii (Sch. Bip.) Drake, Ill. Fl. Ins. Mar. Pac. 209. 1890. **Type.** Marquesas Islands. Nuku Hiva, D.E.S.A. Jardin 41 (holotype: P).
Bidens ahnnei Sherff, Bot. Gaz. (Crawfordsville) 76: 165. 1923. **Type.** Marquesas Islands. Nuku Hiva, Hakaui, December 1916, C. Henry s.n. (holotype: F [452364]!; isotype: F!).
Bidens collina Deg. & Sherff, Bot. Gaz. (Crawfordsville) 96: 144. 1934; Publ. Field Mus. Nat. Hist., Bot. Ser. 16: 79. 1937.
Campylotheca collina (Deg. & Sherff) F. Brown, Bernice P. Bishop Mus. Bull. 130: 354. 1935. **Type.** Marquesas Islands. Hiva Oa: Tehutu, 100 m, 19 May 1929, E.P. Mumford & A.M. Adamson 400 (holotype: NY-00162622!; isotype: BISH!).

##### Description.

Erect subshrubs 0.5–2 m tall. Leaves simple or very rarely (Nuku Hiva) compound and trifoliolate, 3–15.5 cm long including petiole, blades lanceolate to elliptic-lanceolate, 2–11 × 1.2–5 cm, glabrous or lower surface occasionally pubescent, especially on young leaves, margins variably serrate to serrulate or subentire, apex acuminate or occasionally acute or caudate (Nuku Hiva). Heads 10–80(–100+), glabrous to sparsely pubescent at base, in diffuse cymes terminating main stem and lateral branches, main inflorescence axis up to 6.5 cm, heads 2–5 mm in diameter excluding rays, peduncles 0.1–5 cm long, glabrous or sparsely pubescent; outer involucral bracts 1–4.5 mm long, well differentiated from inner bracts, glabrous to sparsely ciliate; ray florets 3–12 per head, sterile, rays yellow, 3–12 × 1–4 mm, apex rounded, entire or bi- or tri-lobed; disk florets ca. 12–25, perfect. Achenes gray to black, straight or slightly curved, 3–8 × 0.5–1 mm, margin setose; pappus of 0–2 short awns. *n* = 36 (Gillett 1972).

##### Distribution.

Marquesas Islands, occurring on Nuku Hiva, Ua Huka, Hiva Oa, Mohotani, and Tahuata, 10–800(–1080) m. The occurrence on Mohotani is based on observation by Jean-François Butaud (pers. comm., 2014), but should be verified by collections.

**Figure 3.**
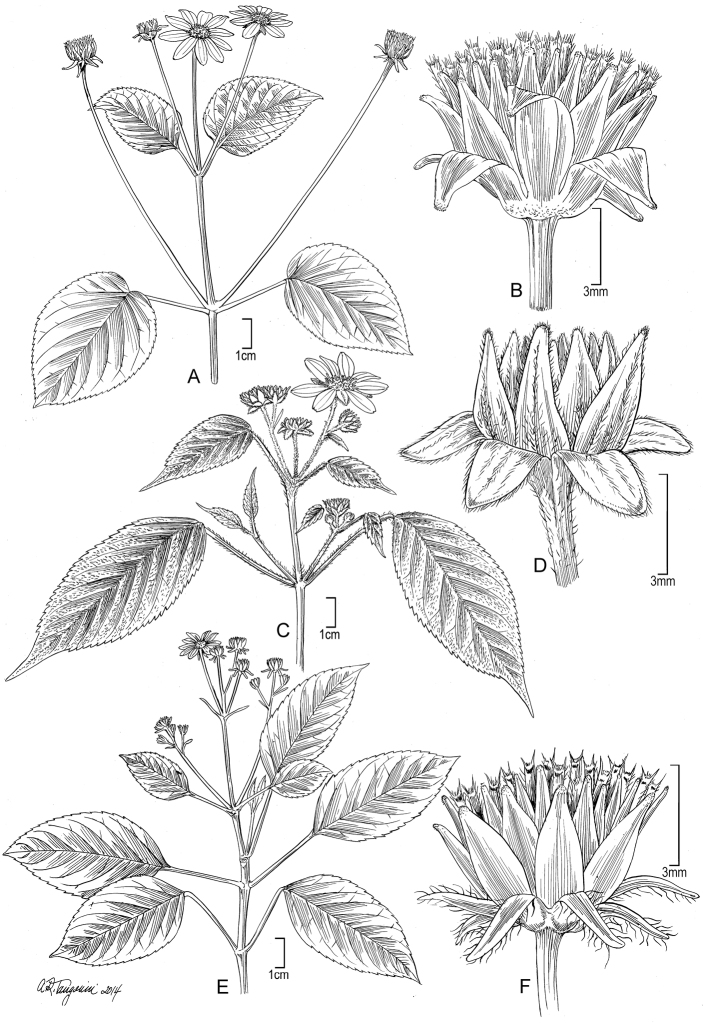
*Bidens beckiana* (F. Br.) Sherff (**A–B**). **A** Distal part of plant **B** Head post-flowering showing involucral bracts and apex of achenes. Drawn from Florence 9368 (US) [A] and Perlman 10064 (US) [B]. *Bidens cordifolia* Sch. Bip. (**C–D**). **C** Distal part of plant **D** Head post-flowering showing involucral bracts. Drawn from Wood & Perlman 4600 (US). *Bidens polycephala* Sch. Bip. (**E–F**). **E** Distal part of plant **F** Head post-flowering showing involucral bracts and apex of achenes. Drawn from Perlman 15878 (US). Image from field shots used to augment illustration of Wood 10504.

##### Habitat.

*Bidens polycephla* is known from slopes and ridges to windswept cliffs, in mesic forests with *Freycinetia impavida* (Gaudich. ex Hombr.) B. C. Stone, *Metrosideros collina*, and *Dicranopteris linearis* (Burm. f.) Underw., or *Hibiscus tiliaceus* L. and *Pandanus tectorius* Parkinson ex Du Roi forest.

##### Conservation status.

Proposed IUCN Red List Category **Endangered** (EN), criteria B2b i–iii): B2, total area of occupancy less than 500 km² (ca. 50 km²); b (i–iii), habitat continuing decline inferred. The suitable habitat for *Bidens polycephla* on Nuku Huka (ca. 340 km²), Ua Huka (ca. 83 km²), and Tahuata (ca. 61 km²) is indicated as an endangered environment, threatened by human activity (deforestation), feral animals, and invasive plants, thus reducing the extent of the forest. However, *Bidens polycephala* has been observed to be locally common in some areas with considerable regeneration, and alternatively it might be considered Near Threatened (NT) (Butaud, pers comm. 2014).

##### Specimens examined.

**Marquesas Islands. Nuku Hiva:** Hakaui, ridge, 800 m, 7 May 1921, Brown 405B (BISH); Between Taiohae Bay and Hooumi Bay, 900 m, 20 Jul 1977, Gagné 1155 (BISH, US), Gagné & Montgomery 1162 (BISH); Terre Deserte, Haatuatua Valley, 1050 m, 1 Aug 1988, Gagné & Montgomery 2463, 2471 (BISH); Route Toovii–Terre Deserte, km 4 après le col, 940 m, 8°50"S/140°10"W, 9 Dec 1982, Florence 4353 (BISH, K, NY, P, US); Route Toovii–Terre Deserte, km 1 après le col, 960 m, 8°52"S/ 140°10"W, 10 Dec 1982, Florence 4390 (BISH, P, PAP); Moyenne Taipivai, sous le captage de la cascade de Teuakueenui, 440 m, 8°51"S/140°6"W, 4 Aug 1987, Florence 8471 (BISH, P, PAP, US); Taipi Vai, 500 m, 7 May 1921, Brown 405A (BISH); Piste Aakapa–Pua, sous le col de Taahui, 260 m, 8°49"S/140°9"W, 7 Aug 1987, Florence 8502 (PAP), 180 m, 8502(PAP); Toovii, face SE du Mt. Tuhokia, 1080 m, 8°50"S/140°8"W, 10 Mar 1986, Florence 7516 (PAP); Moyenne vallée de Hakaui, secteur central, 240 m, 8°54"S/ 140°9"W, 23 Jul 1987, Florence 8387 (BISH, P, PAP); Hakaui Valley, 61 m, 26 Jun 1988, Perlman 10010 (BISH, PAP, PTBG); Toovii, épaulement au-dessus du réservoir, 1020 m, 8°52"S/140°9"W, 7 Dec 1982, Florence 4332 (BISH [2], K, NY, P, US); Route Toovii–Terre Deserte, 2 km après le col, 960 m, 8°52"S/140°10"W, 4 Jun 1984, Florence 6895 (BISH [2], P, PAP); South spur of the summit ridge of the island, between Hakui and the Tapuaooa shelter on the Toovii Plateau, 1070 m, 21 Jul 1970, Gillett 2194 (BISH, US); Mountain near Hakaui, s.d., Sherff 3090 (BISH); 22 Dec 1919, Sherff 3084-b (BISH [2]); 17 Sep 1919, Sherff 3084-a (BISH); Ridge, 800 m, 18 Oct 1922, Quayle 1588 (BISH); Partie S de Toovii, 750 m, 11 Jul 1975, Thibault 143 (BISH, P, US); Taipivai, Teuakueenui Falls area, 351 m, 8°51"2'S/140°6"10'W, 25 Jun 1997, Wood, Meyer & Luce 6356 (BISH, HAST, MO, NY, P, PAP, PTBG, UBC, US). **Ua Huka:** Ridgecrest south of central Vaipae’e Valley, approx. 2 km E of bend, 450 m, 18 Mar 1964, Decker 1877 (BISH, MO, PTBG, US); central crest of island N of Vaipae’e, 550-600 m, 16 Mar 1964, Decker 1868 (US); seacliffs just before the village of Hokatu, road from Hane to Hokatu, 12 m, 1 Jul 1997, Perlman, Wood & Meyer 15878 (MO, NY, P, PAP, PTBG, UBC, US, WU); Hitikau region, ascended via the Matukuoha Ridge over-looking Hane, constitutes the summit of the single crater of Ua Huka, 730 m, 5 Dec 2003, Wood 10495 (PTBG); Hane valley, ridge and cliffs above Tiki marae, back of valley on west side, 518 m, 8°54"50.6'S/138°31"51.5'W, 12 Jun 2004, Perlman, Lorence, Dunn & Wood 19009 (BISH, P, PAP, PTBG, US); Ridgecrest about 1 km N of Tahoatikikau crater, 300 m, 23 Mar 1964, Decker 1918 (BISH, CHR, K, MO, P, US); Hanahouua Valley, back of valley, cliffs near ridge between Hanahouua and Hanaei, 488 m, 8°54"46.7'S/139°30"8.68'W, 28 Jul 2005, Perlman & Meyer 19750 (BISH, MPU, NY, P, PAP, PTBG, US) [used in molecular study, Funk et al.]; Summit of Hitikau, crest of island, at very summit, 853 m, 28 Jun 1997, Perlman, Wood & Meyer 15847 (PTBG); Vaipaee, upper valley, Aunoa property, 60 m, 8°54"40'S/139°34"11'W, 9 Dec 2003, Wood 10504 (AD, BISH, BR, K, MO, NY, P, PAP, PTBG, US); Hokatu village coast, scattered along coast and cliffs from Hokatu to Tenaha to Haavahae, 31 m, 8°55"6.32'S/139°31"17.8'W, 10 Jun 2004, Perlman 19001 (BISH, P, PAP, PTBG, US); Hane/Hokatu cliff zone, 520 m, 11 Dec 2003, Wood & Meyer 10515 (BISH, P, PAP, PTBG, US); Hanahouua valley, back of valley below cliff walls, 1500 ft, 8°54"46.7'S/139°30"8.68'W, 26 Jun 2004, Perlman & Wood 19071 (BISH, P, PAP, NY, PTBG, US); Crest of ridge W of Vaipae’e Valley, approx. 3 km inland from bayhead, 280–300 m, 25 Feb 1964, Decker 1728 (BISH, MO, P, PTBG, US); Matukuoha, on ridge leading to Hitikau, 549 m, 28 Jun 1997, Perlman, Wood & Meyer 15857 (BISH, MO, NY, MPU, OS, P, PAP, PTBG, US). **Tahuata:** Near village of Hapatoni, coast to south of village, on seacliffs about .25–5 miles from Hapatoni, 46 m, 9°58"14.2'S/139°7"8.01'W, 3 Feb 2003, Perlman & Price 18376 (BISH, P, PAP, PTBG, US); Vaitahu, vers la crête d’Uuau, partie basse de la crête, 550 m, 8 Apr 1975, Schäfer 5468 (K, MPU, US); Base of cliff, above slope, about .3 km S. of Hapatoni, 150 m, 3 Feb 2003, Price 218 (BISH, P, PAP, PTBG, US) [used in molecular study, Funk et al.]; Trail from Vaitahu to Hapatoni, Tiana, the Vaitahu water catchment, 190 m, 30 Jan 1975, Oliver & Schäfer 3068 (BISH, CBG, CHR, K, MO, NSW, L, PTBG, US); Hanatetena, main valley, first deep side gulch to the north, 457 m, 2 Feb 2003, Wood 10081 (PTBG, US); Near village of Hapatoni, coast to south of village, on seacliffs about 0.25-5 miles from Hapatoni, 100 m, 9°58"23'S/139°7"8'W, 3 Feb 2003, Perlman & Price 18378 (BISH, NY, P, PAP, PTBG, US).

##### Discussion.

*Bidens polycephala* is the most variable of the Marquesan *Bidens*, and is also the most widespread, occurring on four islands. It is similar to *Bidens henryi* but differs in having much shorter involucral bracts and in the large but variable number of heads among individuals, and it occupies drier and lower elevation habitats. Some of the collections of this species from Nuku Hiva seem to grade toward *Bidens bipontina* as they have more caudate leaf apices and longer rays (e.g., Florence 4332, 4353, and 4390 from Toovii Plateau area) than collections from other areas of Nuku Hiva and other islands. Specimens from the Terre Deserte, Haatuatua Valley (Gagné & Montgomery 2463, 2471) have heads on the smaller end of the variation like some other collections from Nuku Hiva, but uniformly have compound leaves. Compound leaves occurs only in Haatuatua and the adjacent Tapueahu Valleys, and they are found in all three of the species that occur there (*Bidens cordifolia*, *Bidens bipontina*, and *Bidens polycephala*). Otherwise these species all have simple leaves. The only exception to this is the rare occurrence of compound leaves in *Bidens microcephala* on Fatu Hiva. This pattern could be the result of presence of compound leaves in populations of one of the three species and hybridization among them at some time in the past. Two collections are considered putative hybrids and are discussed below. Gillett (1972) used a collection (Gillett 2194) in his experimental studies of Pacific *Bidens*. He determined that *Bidens polycephala* was self-compatible and is capable of forming fully fertile F_1_ hybrids with several Hawaiian species (*Bidens mauiensis* (A. Gray) Sherff, *Bidens menziesii* (A. Gray) Sherff, and *Bidens molokaiensis* (Hillebr.) Sherff).

#### 3. Bidens uapensis

(F. Brown) Sherff, Publ. Field Mus. Nat. Hist., Bot. Ser. 16: 115. 1937.

http://species-id.net/wiki/Bidens_uapensis

Figure 4

Campylotheca uapensis F. Brown, Bernice P. Bishop Mus. Bull. 130: 358. 1935.

##### Type.

Marquesas Islands. Ua Pou: Mt. Tekahoipu, 810 m, 9 September 1922, E.H. Quayle 1149 (lectotype, designated by Sherff, Publ. Field Mus. Nat. Hist., Bot. Ser. 16: 115. 1937: BISH-723226!). An additional syntype is Quayle 1066 (BISH).

##### Description.

Erect, suffrutescent perennial herbs 0.6–3 m tall. Leaves simple, 4.5–12 cm long including petiole, blades lanceolate to lanceolate-elliptic, 3–8.5 × 1–4 cm, mostly glabrous, except for a tuft of hairs on lower surface at base, margins serrulate to serrate, apex acuminate to occasionally acute. Heads 3–12(–16), in diffuse cymes, terminating main stem and lateral branches, 2–8 mm in diameter excluding rays, peduncles 0.4–7 cm long, stout; outer involucral bracts 1.5–8 mm long, well differentiated from inner bracts, margins ciliate, inner bracts often with a tuft of hairs at apex; ray florets 5–11 per head, sterile, rays yellow, 7–15 × 1.5–5 mm; disk florets ca. 23–25, perfect. Achenes gray or black, straight, 3–5 × 0.5 mm, tipped, margin setose; pappus of 2 irregularly antrorse barbed awns.

**Figure 4.**
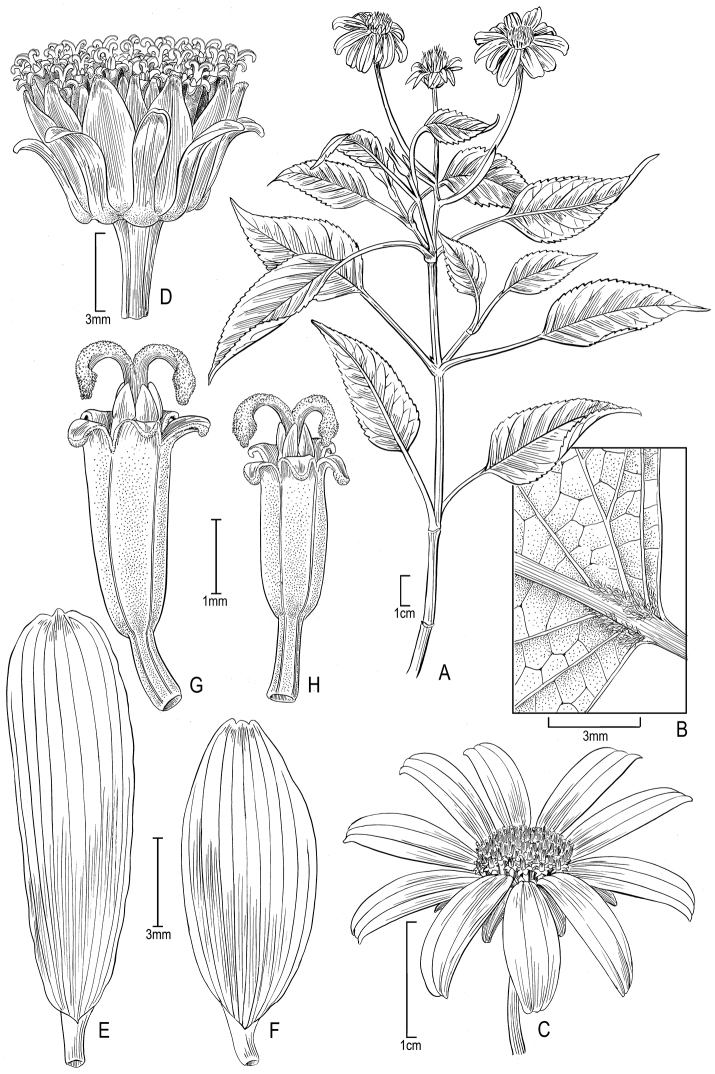
*Bidens uapensis* (F. Brown) Sherff **A** Distal part of plant **B** Leaf surface (underside) **C** Head showing rays and disk florets **D** Head with ray florets removed to show involucral bracts **E–F** Ray florets, showing variation **G–H** Disk florets, showing variation. Drawn from Dunn 241 (US) [**A–E, G**], Perlman & Wood 19083 US [**F, H**].

##### Distribution.

Marquesas Islands, endemic to Ua Pou, 6–920 m.

##### Habitat.

Ridges and slopes in openings of mesic or wet forest, with common species such as *Metrosideros collina*, *Weinmannia marquesana*, *Freycinetia impavida, Hibiscus tiliaceus, Pandanus tectorius*, and *Dicranopteris linearis*.

##### Conservation status.

IUCN Red List Category **Endangered** (EN), criteria B2a, B2b i-iii): B2, total area of occupancy less than 500 km² (c. 50 km²); B2a, fragmented populations; b (i-iii), habitat continuing decline inferred. The suitable habitat for *Bidens uapensis* on Ua Pou (c. 105 km²) is indicated as an endangered environment, threatened by human activity (deforestation, fire), feral animals, and invasive plants, thus reducing the extent of the forest. Nevertheless, *Bidens uapensis* has been observed to be locally common in some areas with good regeneration (Butaud, pers. comm. 2014), and consequently an alternative category might be Least Concern (LC).

##### Specimens examined.

**Marquesas Islands. Ua Pou:** Poumaka (above Hakahetau), SE facing near the base, 675 m, 9°23"38'S/140°4"53'W, 30 Nov 2003, Dunn 318 (BISH, K, MO, NY, PAP, P, PTBG, US); Along road between Hakamaii village and Hakotu valley, 300 m, 9°24"13'S/140°6"52'W, 15 Jul 2003, Lorence, Dunn, Wood & Teikiehuupoo 9098 (BISH, P, PAP, PTBG[2], US) [used in molecular study, Funk et al.]; Teavaiteaki Pass, along road to Hohoi, between Hohoi and Pass, Moukatutai, 274 m, 2 Jul 1997, Perlman, Wood, Meyer, Kautai & Kohumoetini 15883 (BISH, MO, P, PAP, PTBG, UBC, US); Toua, growing alongside a newly cut road at the pass into Hakatau valley, 550 m, 9°26"4'S/140°3"50'W, 16 Jul 2003, Dunn, Lorence & Wood 241 (P, PAP, PTBG, US) [used in molecular study, Funk et al.]; Kuata’u Mountain, near Hakahou, 6 m, 9°21"24'S/140°3"24'W, 3 Jul 1997, Wood 6443 (BISH, MO, P, PAP, PTBG, UBC, US); Hakahau, Tehutu Road, 700 m, 10 Aug 1988, Gagné & Montgomery s.n. (BISH [2]); Teavahaakiti, steep slopes of main ridge to S of Oave, N & E facing cliffs between Teavahaakiti & Tekohepu, 869 m, 5 Jul 1997, Perlman & Wood 15912 (AD, MO, NY, P, PAP, PTBG, US); Teavahaakiti, cliffs between Tekohepu & Teavahaakiti, 686 m, 4 Jul 1997, Perlman, Wood & Kautai 15894 (AD, BISH, HAST, MO, NY, P, PAP, PTBG, UBC, US); Forested ridges and slopes to the north and west of Pouakei, 701 m, 9°23"S/140°5"W, 8–9 Jul 2004, Wood & Perlman 10839 (BISH, P, PAP, PTBG, US); Pouakei and along ridge trail to Pouakei, across valley from Pou Maka and Pou Maka trail, 808 m, 9°23"9.63'S/140°4"9.81'W, 9 Jul 2004, Perlman & Wood 19184 (PTBG, US); Ridge just north of Oave, between Oave and Matahenua, high mountain peaks along main backbone ridge, 945 m, 9°23"45.5'S/140°4"43.3'W, 3 Jul 2004, Perlman & Wood 19083 (BISH, NY, P, PAP, PTBG, US); Hakahai, valle de Hakaohoka, environs du sommet du Mt. Aefiti, 400 m, 9°22"S/140°4"W, Dec 1985, Ottino s.n. (PAP); 1500 m, 7 Sep 1922, Quayle 1066 (BISH); Central Ua Pou including the summit crest regions around Oave and the near-by peak of Matahenua, 899–924 m, 9°23"S/140°4"W, 2 Jul 2004, Wood & Perlman 10820 (PTBG).

##### Discussion.

*Bidens uapensis* can be differentiated from other species in having achenes that have pronounced barbed awns and leaf blades with a tuft of hairs on lower surface at the base, a character not seen in any of the other Marquesas *Bidens*.

#### 4. Bidens microcephala

W.L. Wagner, J.R. Clark & Lorence sp. nov.

urn:lsid:ipni.org:names:77139444-1

http://species-id.net/wiki/Bidens_microcephala

Figure 5

##### Type.

**Marquesas Islands.** Fatu Hiva: Angled rocks above Omoa village (Aiguilles Rocheuses), south-facing, sea cliffs, 524 m [10°31"42.8'S/138°41"17.2'W], 6 September 1995, S. Perlman & K. R. Wood 14946 (holotype: PTBG-066383; isotypes: BISH, K, MO, MPU, NY, P, UC, US).

##### Description.

Erect, suffrutescent perennial herbs 1–1.3 m tall. Leaves simple, (4–)6–14.5(–20) cm long including petiole, rarely compound, blade lanceolate to lanceolate-elliptic, occasionally lanceolate-linear, (1.5–)3.5–9.5(–14) × (0.9–)1.3–4 cm, glabrous, margins serrate to serrulate, occasionally subentire, apex acuminate. Heads 40–50, in compact cymes, becoming diffuse at maturity, heads often in tight clusters, terminating main stem and lateral branches, main inflorescence axis 6–9 cm, heads 1–2 mm in diameter, peduncles 0.1–1.5 cm long, glabrous; outer involucral bracts 1–2 mm long, well differentiated from inner bracts; ray florets absent; disk florets (1–)5–10, perfect. Achenes gray or black, straight, 3–4 × 0.5 mm, sparsely setose; pappus absent.

**Figure 5.**
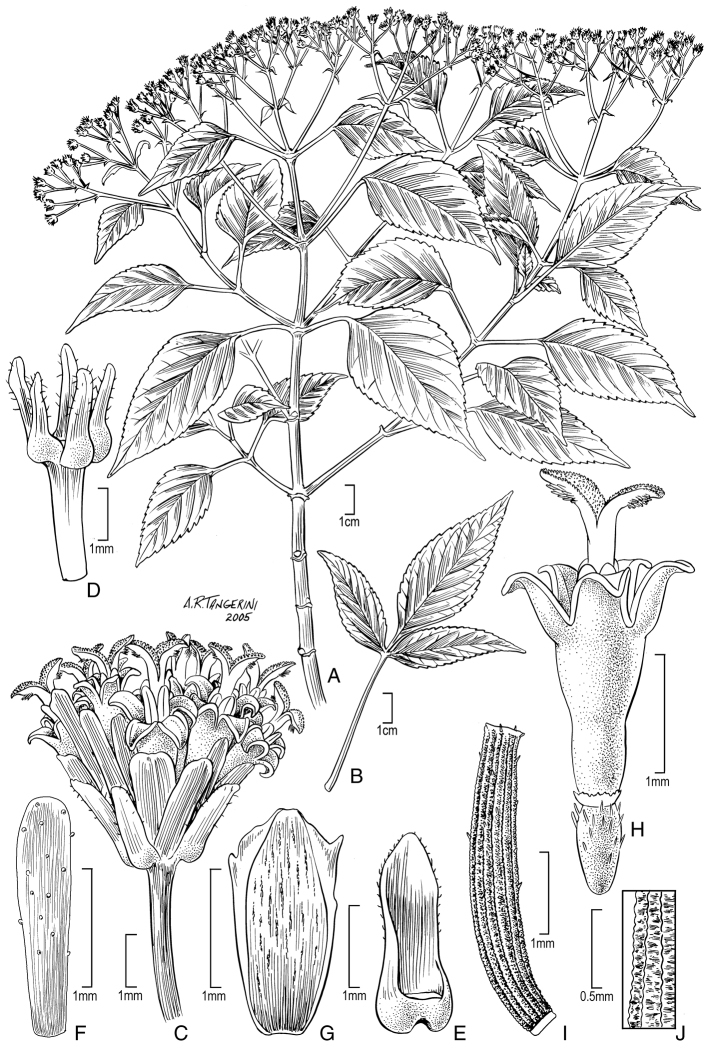
*Bidens microcephala* W.L. Wagner, J.R. Clark & Lorence **A** Distal part of plant **B** Compound leaf **C** Head showing bracts and disk florets **D** Outer Involucral bracts and peduncle **E** Outer involucral bract showing basal gibosity **F** Pale **G** Inner involucral bract **H** Disk floret **I** Achene **J** Inset showing achene surface. Drawn from Perlman 18444 (PTBG) [**A, C, D, G** and **H**], Perlman 18416 (unmounted) [**B**], pickled material of PTBG cult. 950676 (from Perlman & Wood 14996) [**E, I and J**], and Wood 10103 [**F**].

##### Distribution.

Marquesas Islands, endemic to southern and central Fatu Hiva, 10–625 m.

##### Habitat.

On sea cliffs in mixture of native shrub and trees and alien vegetation, with native species such as *Dodonaea viscosa* Jacq., *Hibiscus tiliaceus*, *Pisonia grandis* R. Br., *Celtis pacifica* Planch., *Psydrax odorata* (G. Forst.) A.C. Sm. & S.P. Darwin, *Wikstroemia coriacea* Seem., and *Dinebra xerophila* (P.M. Peterson & Judz.) P.M. Peterson & N. Snow, and naturalized species like *Casuarina equisetifolia* L., *Coffea arabica* L., *Cordyline fruticosa* (L.) A. Chev., *Psidium guajava* L., *Miscanthus floridulus* (Labill.) Warb., and *Morinda citrifolia* L.

##### Etymology.

The specific epithet refers to the extremely small heads, probably the smallest in the genus.

##### Conservation status.

Proposed IUCN Red List Category **Critically Endangered** (CR), criteria B1ab, B2a,b (i–iii): B1, total extent of occurrence less than 100 km² (ca. 85 km²), a, severely fragmented and b, continuing habitat decline inferred; B2a, estimated area of occupancy estimated to be less than 10 km²; B2b (i–iii), habitat continuing decline inferred. The estimated area of occupancy for *Bidens microcephala* on Fatu Hiva (ca. 85 km²) is indicated as an endangered environment, threatened by human activity (deforestation and fire), feral animals, and invasive plant species, thus reducing the extent of the suitable habitat. It is still locally common and regenerating on some cliff areas inaccessible to goats, however, so VU might be an alternative category (Butaud, pers. comm. 2014).

##### Specimens examined.

**Marquesas Islands. Fatu Hiva:** Omoa, crete a l’Est du Mt Tefatutea, 625 m, 10°31"S/138°40"W, 25 Jul 1988, Florence & Perlman 9566 (P, PAP); On seacliffs of Aiguilles Rocheuses, S of Omoa village, above Tataaihoa and Tahaoa, 524 m, 6 Sep 1995, Perlman & Wood 14948 (BISH, MPU, P, PAP, PTBG, US) [used in molecular study, Funk et al.]; Tetio, seacliffs between Omoa and Hanavave, Tetio lies S of Matatu Point, about halfway to Omoa, 6–110 m, 15 Sep 1995, Perlman & Wood 14996 (BISH, MO, P, PAP, PTBG, US), cultivated plant from seed from this source, 5 Nov 1996, Lorence 7823 (BISH, PTBG, UBC, US); Hanavave Valley, cliffs below Teani, near Teeavahinenao Pass, up Uiha stream to back wall, 378 m, 16 Sep 1995, Perlman, Wood & Alexi 15009 (BISH, MO, MPU, NY, PAP, PTBG, US); Aiguilles Rocheuses, Tataaihoa side, 533-579 m, 6 Sep 1995, Wood & Perlman 4465 (BISH, K, MO, MPU, NY, P, PTBG, US); Tetio, S of Hanavave, 31-152 m, 15 Sep 1995, Wood & Perlman 4539 (AD, BISH, MO, MPU, NY, P, PAP, PTBG[2], US); About 1 km S of Omoa, 10 m, 31 Oct 1974, Decker 2689 (BISH, BKL, CBG, CHR, K, L, MO, MPU, NSW, NY, P, PAP, PTBG, US); Ridge to south of Omoa village at Angled Rocks (Aguilles Rocheuses), 585 m, 10°31"42.8'S/138°41"17.2'W, 11 Feb 2003, Perlman 18416 (BISH, P, PAP, PTBG, US); Coastline between Hanaui to Hanahoua, north of Omoa, 9 m, 10 Feb 2003, Wood & Price 10103 (BISH, MPU, PTBG, OS, US); Ouia valley, back of valley on south side in forest, 210 m, 10°29"10.3'S/138°37"12.9'W, 14 Feb 2003, Perlman 18444 (BISH, MPU, P, PAP, PTBG, US); Ridge from Omoa to Aiguilles Rocheuses, along ridge crest to Tefatutea Peak, sea cliffs of Needle Rocks above Tahaoa, 594 m, 22 Jul 1988, Perlman 10156 (AD, BISH, MO, MPU, P, PAP, PTBG, US) ; about 1 km along coast N. of Hanaui, 10 m, 10 Feb 2003, Price & Wood 231 (PAP, PTBG, US) [used in molecular study, Funk et al.]; Chemin d'Omoa à Hanavave, au-dessus de la vallée de Teatapu, 550 m, 18 Sep 1975, Schäfer 5747 (BISH, MPU, P, PAP, PTBG, US).

##### Discussion.

*Bidens microcephala* exhibits the most reduced heads of all Marquesan *Bidens*, generally having 10 or fewer disk florets per head and completely lacking in ray florets. In several specimens (Price & Wood 231, Wood & Perlman 4539, Wood & Price 10103, Perlman & Wood 14996), this reduction is even more extreme; flower heads in these specimens contain consistently fewer than 4 florets each, often only two or even occasionally one. These latter specimens also universally exhibit entire to subentire leaf margins and might represent yet another evolutionary lineage worthy of formal recognition (e.g., in Lorence 7823, specimen from cultivated plant grown from seed from Perlman 14996).

#### 5. Bidens woodii

W.L. Wagner, J.R. Clark & Lorence sp. nov.

urn:lsid:ipni.org:names:77139445-1

http://species-id.net/wiki/Bidens_woodii

Figure 6

##### Type.

Marquesas Islands. Ua Pou: Central Ua Pou including the summit crest regions around Oave and the nearby peak of Matahenua, 899–924 m, 9°23"S/140°4"W, 2 Jul 2004, Wood & Perlman 10806 (holotype: US-3547652; isotypes: BISH, P, PAP, PTBG) [used in molecular study, Funk et al.].

##### Description.

Erect, suffrutescent perennial herbs 0.5–1 m tall. Leaves simple, 5–7 cm long including petiole, petiole stout, blades thick and coriaceous, ovate to broadly ovate, 3–4.5 × 1.8–2.5 cm, glabrous, the veins on lower surface conspicuously raised, margins serrate, apex short-acuminate. Heads 1–2, in compact cymes terminating main stem and lateral branches, 4–5 mm in diameter excluding rays, peduncles ca. 2 cm long, stout, glabrous; outer involucral bracts ca. 3–4 mm long, broad, well differentiated from inner bracts; inner bracts dark purple, erose and with a few short hairs; ray florets ca. 6–8, sterile, rays yellow, obovate, 10–12 × 4–7 mm, apices often bi- or tri-lobed; disk florets ca. 30–34, perfect, corollas yellow; pappus absent. Achenes not seen.

**Figure 6.**
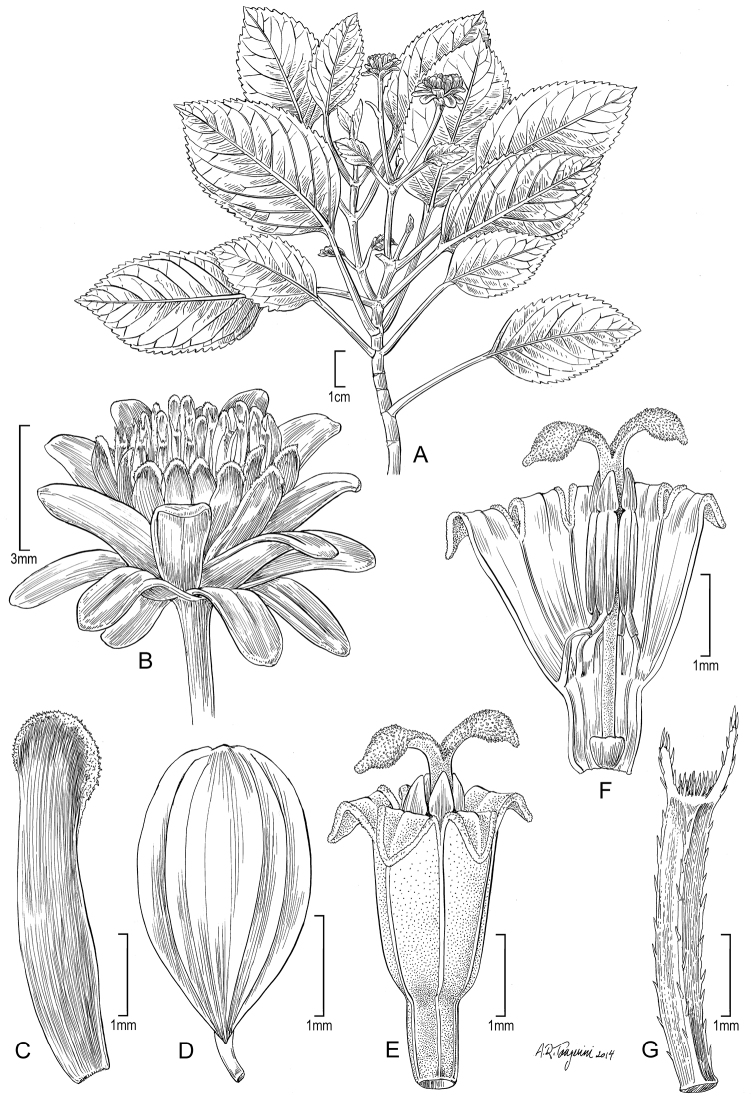
*Bidens woodii* W.L. Wagner, J.R. Clark & Lorence **A** Habit **B** Head showing involucral bracts, pales and achene tips **C** Pale **D** Ray corolla **E** Disk floret (without ovary) **F** Disk corolla longitudinal section **G** Achene. Drawn from Wood & Perlman 10806 (holotype, US).

##### Distribution.

Marquesas Islands, Ua Pou, known only from the type on the summit crest around Oave and the nearby peak of Matahenua, ca. 920 m.

##### Habitat.

Occurs in low shrubland of *Metrosideros collina* with *Dicranopteris linearis* on the windswept and cloud-shrouded summit area.

##### Etymology.

This new species is named for Kenneth R. Wood, who made the only known collection of it and who has contributed greatly to our knowledge of the flora of the Marquesas and Hawaii through his collections, excellent photography, and field observations.

##### Conservation status.

IUCN Red List Category **Endangered** (EN), criteria B2a, B2b i-iii: B2, total area of occupancy less than 500 km² (c. 50 km²); B2a, fragmented populations; b (i-iii), habitat continuing decline inferred. The suitable habitat for *Bidens woodii* on Ua Pou (c. 105 km²) is indicated as an endangered environment, threatened by human activity (deforestation, fire), feral animals, and invasive plants, thus reducing the extent of the suitable habitat.

##### Discussion.

*Bidens woodii* is most similar to *Bidens evapelliana* but can be differentiated by its habit (erect versus prostrate, and 0.5–1 m versus 0.3–0.5 m tall stem), leaves with raised venation, and obovate rays. It is known only from the type collection.

#### 6. Bidens evapelliana

W.L. Wagner, J.R. Clark & Lorence sp. nov.

urn:lsid:ipni.org:names:77139446-1

http://species-id.net/wiki/Bidens_evapelliana

Figures 7 , 8

##### Type.

Marquesas Islands. Fatu Hiva: Slopes of Mounanui, on open ridge, 10°28'656"S/138°38'149"W, 730 m, 16 Jul 2005, Perlman 19665 (holotype: PTBG-066395; isotypes: BISH, MO, P, US).

##### Description.

Prostrate, suffrutescent perennial herbs up to 0.3–0.5 m tall. Leaves simple, coriaceous, 1.3–3.6(–7) cm long including petiole, petiole relatively short, blades elliptic to ovate, 1–2.8(–5.6) × 0.7–2(–3.5) cm, glabrous, margins serrate, apex rounded to acute. Heads 1–3, in compact cymes terminating main stem and lateral branches, 4–8 mm in diameter excluding rays, peduncles 1–2.5 cm long, glabrous; outer involucral bracts 4–6 mm long, broad, well differentiated from inner bracts; ray florets normally 3–6 per head, sterile, rays yellow, ca. 8 × 2–5 mm; disk florets ca. 23–25, perfect. Achenes tawny to gray, straight, ca. 5 × 1 mm, slightly keeled, glabrous; pappus of 2 irregular antrorse barbed awns.

**Figure 7.**
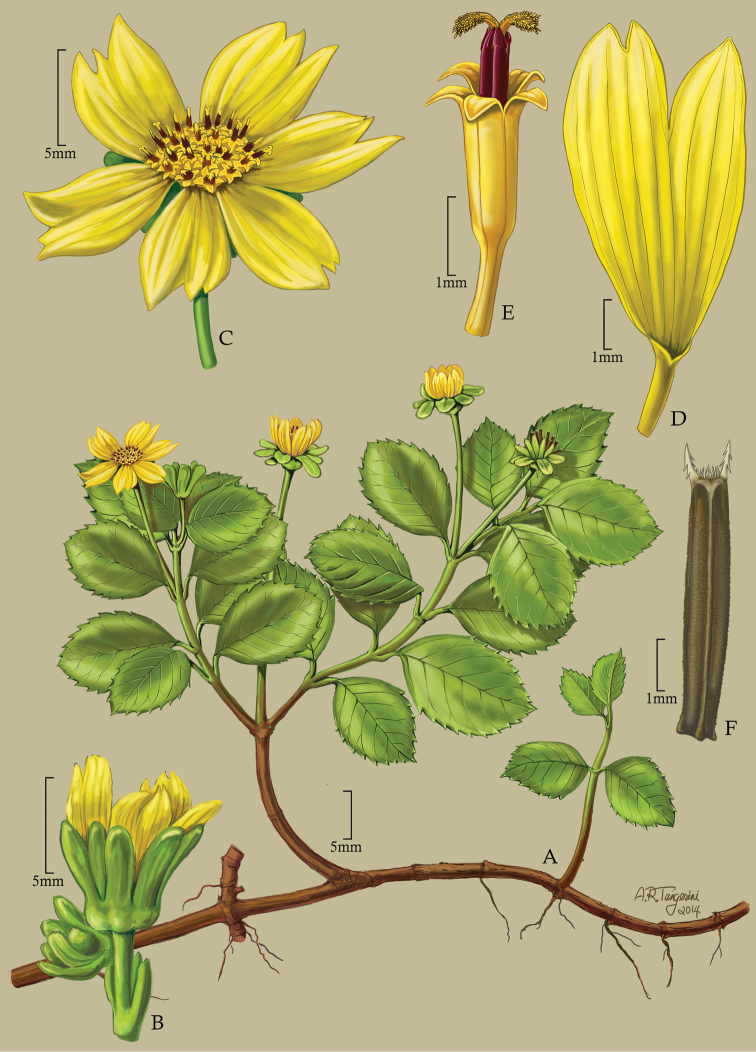
*Bidens evapelliana* W.L. Wagner, J.R. Clark & Lorence **A** Habit **B** Young head showing pre-flowering florets **C** Head showing ray and disk florets **D** Ray corolla **E** Disk corolla **F** Achene. Drawn from Perlman 19665 (isotype, US) and field images of collection [**A, B**], and Florence & Perlman 9590 (US) [**C–F**].

##### Disribution.

Marquesas Islands, known only from the south-central part of Fatu Hiva from Mt. Natahu and Teavapuhiau Pass to Mounanui and Touaouoho, 700–850 m.

##### Habitat.

Scattered on ridgetops and cliffs, in scrubland or low forest of *Metrosideros collina*, *Freycinetia impavida*, *Crossostylis biflora* J.R. Forst. & G. Forst., *Hibiscus tiliaceus*, *Pandanus* sp., *Weinmannia marquesana*, and *Dicranopteris linearis*.

##### Etymology.

This species is named in honor of Eva Pell, Under Secretary for Science at the Smithsonian Institution upon her retirement. She served in this position from 2010 to 2014 during which time she worked to strengthen science across the institution. Her accomplishments include creation of the four “Grand Challenges Consortia,” which have been instrumental in developing interdisciplinary collaborations across the Smithsonian. She also played a key role in creating the Smithsonian’s Tennenbaum Marine Observatories Network, the first worldwide network of coastal field sites to standardize measurements of biological change in marine environments.

##### Conservation status.

Proposed IUCN Red List Category: **Critically Endangered** (CR), criteria B1ab, B2a,b (i–iii): B1, total extent of occurrence less than 100 km² (less than 10 km²); a,b, known from a single location; B2a, area of occupancy estimated to be less than 10 km²; B2b (i–iii), habitat continuing decline inferred. The estimated area of occupancy for *Bidens evapelliana* on Fatu Hiva (ca. 85 km²) is indicated as an endangered environment, threatened by human activity (deforestation and fire), feral animals (goats), and invasive plant species, thus reducing the extent of the forest.

##### Specimens examined.

**Marquesas Islands. Fatu Hiva:** Along base of Mt. Natahu, 828 m, 1–3 Aug 1977, Gagné 1266 (BISH); Teavapuhiau Pass, above Ouia Valley, W of pass, 700 m, 1–3 Aug 1977, Gagné 1252 (BISH); Mt. Touaouoho, on NW side of peak, along ridges between Touaouho and Teavapuhiau, 2650 ft [808 m], 8 Sep 1995, Perlman & Wood 14959 (PTBG, US); Crete Ouest du Mt. Mounanui, 810 m, 10°28"S/138°37"W, 26 Jul 1988, Florence & Perlman 9590 (BISH, P, PTBG, US); slopes of Mounanui above Vaieenui Falls, on ridge top, below Mounanui, 2500 ft [760 m], 26 Jul 1988, Perlman & Florence 10166 (AD, BISH, F, MO, NY, OS, P, PAP, PTBG, TEX, UBC, US); on ridges west side of Mounanui, on ridge leading up from Cascade, 2400 ft [732 m], 10 Sep 1995, Perlman 14977 (BISH, P, PAP, PTBG, US); slopes of Mounanui above Vaieenui Falls, on ridge top, below Maunanui, 2780 ft [847 m], 26 Jul 1988, Perlman & Florence 10173 (BISH).

##### Discussion.

*Bidens evapelliana* is distinctive in its nearly exclusive prostrate habit and relatively small elliptic to ovate leaves. One specimen, Perlman 14959, is somewhat atypical to other collections of *Bidens evapelliana* in that it has larger leaf blades 4.3–5.6 × 2.0–3.5 cm, petioles up to 2 cm long, and slightly longer involucral bracts (ca. 6 mm).

#### 7. Bidens wichmanii

W.L. Wagner, J.R. Clark & Lorence sp. nov.

urn:lsid:ipni.org:names:77139680-1

http://species-id.net/wiki/Bidens_wichmanii

Figure 9

##### Type.

Marquesas Islands. Fatu Hiva: Tevaiua, southern summit region, 870 m, 15 Feb 2003, K. R. Wood 10140 (holotype: PTBG-043994; isotypes: AD, BISH, K, MO, MPU, NY, P, PAP, US).

##### Description.

Scandent to ascending suffrutescent perennial herbs 0.5–2 m tall. Leaves simple, 3.5–13.5 cm long including petiole, blades lanceolate to elliptic-lanceolate, 2.3–9.5 × 1.2–4.5 cm, glabrous, margins serrate, apex acute. Heads 1–3, in diffuse cymes terminating main stem and lateral branches, 8–15 mm in diameter excluding rays, peduncles (2–)3.5–14 cm long, glabrous; outer involucral bracts 8–12 mm long, broad, well differentiated from inner bracts; ray florets 14–20+ per head, sterile, rays yellow, 10–18 × 3–4 mm; disk florets ca. 50, perfect, corollas yellow. Achenes black, straight, 7–10 × 1–2 mm, slightly keeled, glabrous; pappus of 2 irregular antrorse barbed awns.

**Figure 8.**
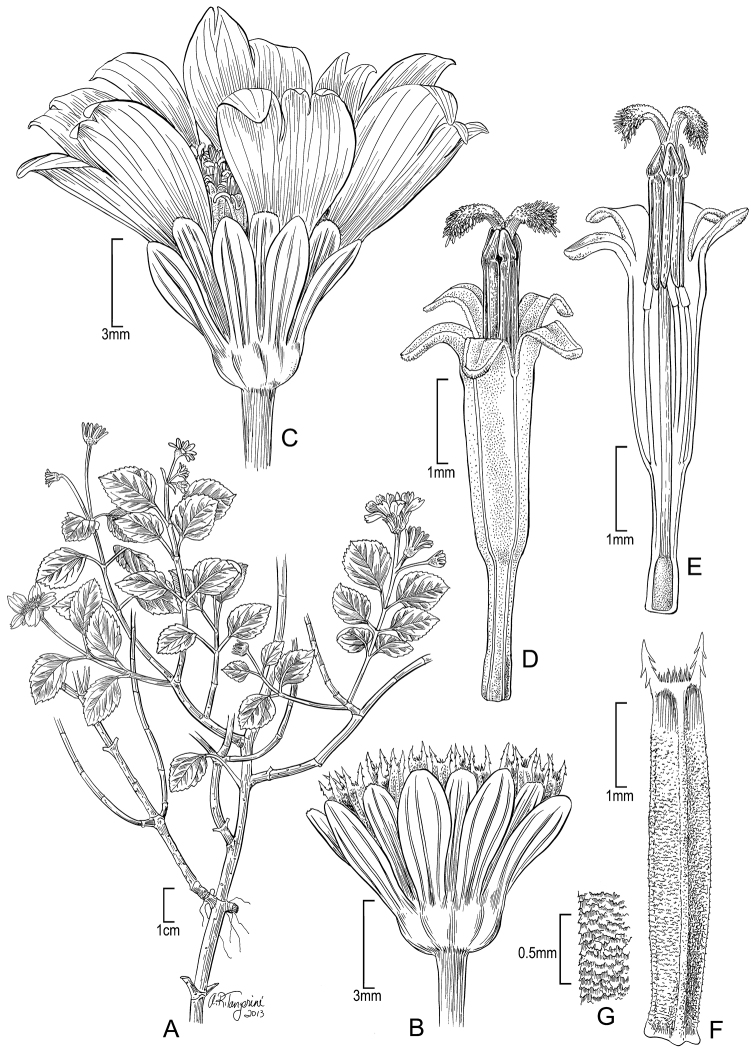
*Bidens evapelliana* W.L. Wagner, J.R. Clark & Lorence **A** Habit **B** Head post-flowering showing involucral bracts and apex of achenes **C** Young head showing immature rays and involucral bracts **D** Disk corolla **E** Disk corolla longitudinal section **F** Achene **G** Inset showing achene surface. Drawn from Perlman 10166 (PTBG) [**A**], Perlman 19665 (isotype, US) [**B**], and Florence & Perlman 9590 (US) [**C–G**].

**Figure 9.**
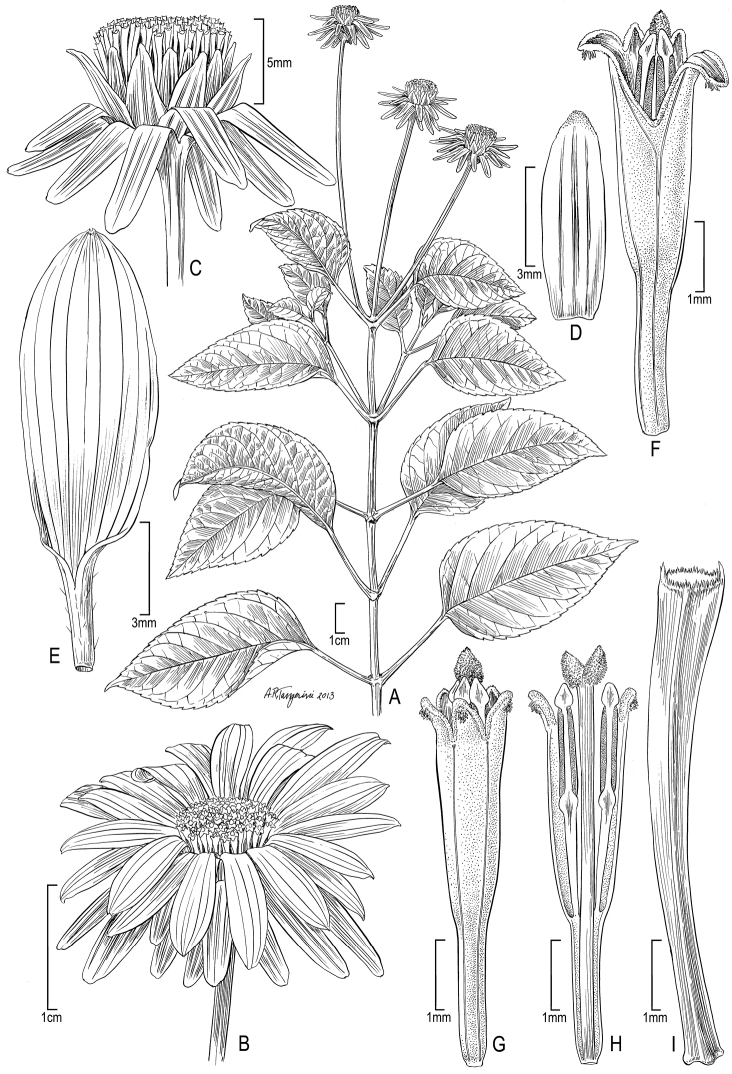
*Bidens wichmanii* W.L. Wagner, J.R. Clark & Lorence **A** Distal part of plant **B** Head showing rays and involucral bracts (outer series) **C** Head post-flowering showing involucral bract **D** Inner involucral bract **E** Ray corolla **F–G** Disk corolla **H** Disk corolla longitudinal section **I** Achene. Drawn from Wood 10140 (isotype, US) [**A, C, I**], and Perlman 10152 (US) [**B, D–G, H**].

##### Distribution.

Marquesas Islands, known only from the central summit region of Fatu Hiva (Tevaiua, Hanativa, and Tekau), 670–870 m.

##### Habitat.

Occurs in shrubland of *Metrosideros collina* with *Freycinetia impavida, Crossostylis biflora*, and *Dicranopteris linearis*, on the windswept summit or near-summit areas.

##### Etymology.

This new species is named for Charles R. “Chipper” Wichman, Jr., Chief Executive Officer and Director of the National Tropical Botanical Garden since 2005, and previously Director of NTBG’s Limahuli Garden on Kaua`i since 1994.

##### Conservation status.

Proposed IUCN Red List Category **Critically Endangered** (CR), criteria B1ab, B2a,b (i–iii): B1, total extent of occurrence less than 100 km² (less than 10 km²); a,b, known from a single location; B2a, area of occupancy estimated to be less than 10 km²; B2b (i–iii), habitat continuing decline inferred. The estimated area of occupancy for *Bidens wichmanii* on Fatu Hiva (ca. 85 km²) is indicated as an endangered environment, threatened by human activity (deforestation and fire), feral animals, and invasive plant species, thus reducing the extent of the suitable habitat.

**S**

##### pecimens examined.

**Marquesas Islands. Fatu Hiva:** Along trail between Omo’a and Uia within 1 km from pass over central ridge, 750 m, 12 Oct 1974, Decker 2397 (BISH, P, NSW, US); Sentier d’Ouia vers Omoa, crête au-dessus de la vallée d’Ouia, S du col de Teava, 680 m, 23 Sep 1975, Schäfer 5843 (MPU[7], PTBG, US); Omoa Valley, past Hoopu to ridge near Tevaiua, top of ridge, 2200 ft [671 m], 22 Jul 1988, Perlman 10152 (BISH, PAP, US); north of Hanativa Valley along ridge crest approaching summit area, 800–900 m, 23 Oct 1974, Decker 2626 (BISH, MO, PTBG, US); along summit crest of ridge above Omoa valley, south of Tekau peak, north of Tevaiua, 760 m, 10°31"10.6'S/138°38"8'W, 15 Feb 2003, Perlman 18453 (PTBG, US); Tevaiua, southern summit region, 870 m, 15 Feb 2003, Wood 10138 (PTBG, US), Wood 10139 (PTBG) [used in molecular study, Funk et al.].

##### Discussion.

*Bidens wichmanii* has the largest heads among the Marquesan species. The glabrous achenes are also distinctive in being keeled along the long axis.

#### 8. Bidens bipontina

Sherff, Bot. Gaz. (Crawfordsville) 85: 10. 1928.

http://species-id.net/wiki/Bidens_bipontina

Figure 2A–C

Bidens serrulata Sch. Bip., Flora 39: 361. 1856, non (Poir.) Desf. 1815.
Coreopsis serrulata (Sch. Bip.) Drake, Ill. Fl. Ins. Mar. Pac.: 210. 1890, non Poiret 1811.
Campylotheca serrulata (Sch. Bip.) F. Br., Bernice P. Bishop Mus. Bull. 130: 356. 1935.

##### Type.

Marquesas Islands. Nuku Hiva, D.E.S.A. Jardin 132 (holotype: P).

##### Description.

Erect, suffrutescent perennial herbs 1–1.3 m tall. Leaves membranous, simple or compound and trifoliolate, 4.3–15 cm long including petiole, blades elliptic-lanceolate to narrowly elliptic, 3–10.5 × 1–3 cm, glabrous, margins serrate, apex long-acuminate to caudate. Heads 6–15, often with two or three heads in tight clusters, in cymes terminating main stem and lateral branches, 2–5 mm in diameter excluding rays, peduncles 2–25 mm long, glabrous; outer involucral bracts ca. 5 mm long, linear, well differentiated from inner bracts, glabrous; ray florets usually 7–9 per head, sterile, rays yellow, 13–20 × 3–4 mm, apex often minutely bi- or tri-lobed or rounded; disk florets ca. 30–32, perfect. Achenes black, 6–7 mm long, margin setose; pappus usually of 2 minute awns.

##### Distribution.

Marquesas Islands, Nuku Hiva, endemic to the Toovii Plateau area, especially Tapueahu Valley, 500–1165 m.

##### Habitat.

*Bidens bipontina* is known only in montane wet shrubland with *Freycinetia impavida* and *Hibiscus tiliaceus* dominant.

##### Conservation status.

Proposed IUCN Red List Category **Endangered** (EN), criteria B2b i–iii): B2, total area of occupancy less than 500 km² (ca. 50 km²); b (i–iii), habitat continuing decline inferred. The suitable habitat for *Bidens bipontina* on Nuku Huka (ca. 340 km²) is indicated as an endangered environment, threatened by human activity (deforestation), feral animals, and invasive plants, thus reducing the extent of the suitable habitat.

##### Specimens examined.

**Marquesas Islands. Nuku Hiva:** Terre Deserte, crête W du Mt. Akaupe, 1165 m, 8°52"S/140°10"W, 10 Aug 1987, Florence 8539 (BISH [2]); Route plateau de Toovii–Terre Deserte, 2 km après le col, 930 m, 8°52"S/140°9"W, 9 Dec 1982, Florence 4348 (BISH, P, PAP, US); Toovii, Ooumu area, top of Tapueahu Valley off new hwy, 1067–1128 m, 8°51"S/140°19"W, 20-22 Sep 1995, Wood & Perlman 4601, (BISH, P, PAP, PTBG, UBC, US); Along old Airport road, across summit crest from Toovii, back drainages of Tapueahu Valley, 1085 m, 21 Sep 1995, Perlman & Wood 15028 (AD, BISH, F, MO, P, PAP, PTBG, US); Ridge crest 2 valleys S of Airport road, back of Tapueahu gulch, to NW of Toovii over summit crest, 1024 m, 21 Sep 1995, Perlman & Wood 15021 (AD, BISH, MO, P, PAP, PTBG) [apparently mixed collection with US sheet, a hybrid]; 500 m, 15 Oct 1922, Quayle 1235 (BISH).

##### Discussion.

*Bidens bipontina* is distinctive in its conspicuously caudate leaves and erect linear involucral bracts.

#### 9. Bidens cordifolia

Sch. Bip., Flora 39: 36. 1856.

http://species-id.net/wiki/Bidens_cordifolia

Figure 3C–D

Coreopsis cordifolia (Sch. Bip.) Drake, Ill. Fl. Ins. Mar. Pac.: 208. 1890.
Campylotheca cordifolia (Sch. Bip.) F. Br., Bernice P. Bishop Mus. Bull. 130: 357. 1935.

##### Type.

Marquesas Islands. Nuku Hiva, D.E.S.A. Jardin 199 (holotype: P).

##### Description.

Erect subshrubs 0.4–1 m tall; young stems and leaves moderately to densely pubescent. Leaves simple or compound and trifoliolate, 6–16 cm long including petiole, blade elliptic to elliptic-lanceolate, 4–9 × 2–4.8 cm, moderately pubescent, especially on lower surface, midrib, veins, and in a line along the petiole, margins serrate, apex acuminate to caudate. Heads usually 5, in cymes terminating main stem and lateral branches, 5–10 mm in diameter excluding rays, peduncles 1.5–4 cm long, sparsely pubescent, hair becoming denser near the heads; outer involucral bracts 5–9 mm long, oblong-elliptic, spreading, well differentiated from inner bracts, moderately to sparsely ciliate; ray florets usually 6–13 per head, sterile, rays yellow, ca. 15 × 4 mm; disk florets ca. 30, perfect. Achenes black, straight, ca. 3 × 0.5 mm, margins setose; pappus usually of 2–3 irregular antrorse barbed awns.

##### Distribution.

Marquesas Islands, occurring only in a relative small area of the Toovii Plateau on Nuku Hiva, from 750–1130 m.

##### Habitat.

*Bidens cordifolia* is known from montane wet shrubland or low forest with *Metrosideros collina* and *Weinmannia marquesan*a with fern understory along with other shrubs and trees such as species of *Coprosma*, *Crossostylis*, *Cyrtandra*, *Dicranopteris*, *Geniostoma*, and *Myrsine*.

##### Conservation status.

Proposed IUCN Red List Category **Critically Endangered** (CR), criteria B1ab, B2a,b (i–iii): B1, total extent of occurrence less than 100 km² (less than 10 km²); a,b, known from a single location; B2a, area of occupancy estimated to be less than 10 km²; B2b (i–iii), habitat continuing decline inferred. The suitable habitat for *Bidens cordifolia* on Nuku Huka (ca. 340 km²) is indicated as an endangered environment, threatened by human activity (deforestation, fire), feral animals (goats), and invasive plants, thus reducing the extent of the forest.

##### Specimens examined.

**Marquesas Islands.** **Nuku Hiva:** Toovii and road from Taiohae Bay to Toovii, 750 m, 3 Aug 1988, Wagner & Lorence 6258 (BISH, US); Toovii, Ooumu area, top of Tapueahu Valley off new hwy, 1067–1128 m, 8°51"S/140°19"W, 20–22 Sep 1995, Wood & Perlman 4600 (BISH, P, PAP, PTBG, US).

##### Discussion.

*Bidens cordifolia* is one of the least known species of *Bidens* in the Marquesas Islands. We have seen only two collections we refer to this species as well as images taken by Jean-François Butaud in 2008 and 2009 of additional populations. In addition to these collections and observations this species was only known from the type and a collection made in 1840 (Barclay 3213, BM) as cited by Sherff (1937).

#### 10. Bidens beckiana

(F. Br.) Sherff, Publ. Field Mus. Nat. Hist., Bot. Ser. 16: 80. 1937.

http://species-id.net/wiki/Bidens_beckiana

Figure 3A–B

Campylotheca beckiana F. Br., Bernice P. Bishop Mus. Bull. 130: 359, fig. 66. 1935.

##### Type.

Marquesas Islands. Eiao, 20 September 1922, R.H. Beck 1529 (holotype: BISH-723228!; isotype: BISH).

##### Description.

Erect subshrubs 0.7–1.3 m tall. Leaves simple, 1.5–9.5 cm long including petiole, blades narrowly ovate or lanceolate, 1–7 × 0.8–4 cm, glabrous or pubescent toward the base and in a line along the petiole, margins serrulate, apex acuminate. Heads (1–)3(–4), in diffuse cymes terminating main stem and lateral branches, 5–15 mm in diameter excluding rays, peduncles 1.5–11.8 cm long, glabrous; outer involucral bracts 4–9 mm long, broad and tapering somewhat at the base, apex sometimes erose, inner bracts sometimes erose at apex; ray florets 5–11, sterile, rays yellow, 9–15 × 2–3 mm wide, apex entire or divided into 2–3 acute to rounded lobes; disk florets ca. 30–34, perfect, corollas yellow. Achenes gray, straight or slightly curved, 8–10 × 1 mm, conspicuously setose; pappus of 2 antrorse barbed awns.

##### Distribution.

Marquesas Islands, occurring on Eiao and Hatutaa, 150–500 m.

##### Habitat.

*Bidens beckiana* is scattered to locally common on steep slopes and ridges, ravines, or cliffs, from mesic to xeric shrublands and grasslands with *Dinebra xerophila*, *Waltheria indica* L., *Cordia lutea* Lam., *Dodonaea viscosa*, and *Thespesia populnea* (L.) Sol. ex Corrêa. Judging from the number of collections *Bidens beckiana* appears to be more common on Hatutaa than Eiao, which has populations of feral sheep, pigs, cats, and rats.

##### Conservation status.

**Endangered** (EN), criteria B2b (i–iii): B2, total area of occupancy less than 500 km² (ca. 47 km²); b (i–iii), habitat quality continuing decline inferred. The suitable habitat for *Bidens beckiana* on Eiao (40 km²) and Hatutaa (7 km²) is indicated as an endangered environment, threatened by feral animals, and invasive plants, thus reducing the extent of the suitable habitat.

##### Specimens examined.

**Marquesas Islands. Eiao:** North side of large valley which is south of Vaituha Valley, Opituha Valley, 320 m, 7 Jul 1988, Perlman & Florence 10051 (BISH, E, F, K, LE, MO, P, PAP, PTBG, OS, UBC, US); NW side of island, Vaituha Bay and summit ridge of island, 400 m, 1 Aug 1977, Gagné 1294 (BISH, US); Tohuanui, secteur est, 480 m, 8°0"S/140°41"W, 8 Jul 1988, Florence & Teikiteetini 9368 (BISH, CHR, K, P, PAP, PTBG, US); Tetuaenui, falaise de la crete, 500 m, 5 Aug 1987, Thibault 1083 (PAP). **Hatutaa:** Plateau, secteur centrale, 320 m, 7°55"S/140°34"W, 11 Jul 1988, Florence & Teikiteetini 9419 (BISH, P, PAP, US), 9426 (BISH, P, PAP, US); Plateau centrale, 150-200 m, 19 Aug 1975, Thibault 148 (BISH, US); Plateau, secteur Ouest, 280 m, 7°56"S/140°35"W, 10 Jul 1988, Florence & Perlman 9408 (BISH, CHR, P, PAP, PTBG, US); Plateau, secteur Ouest, 280 m, 7°56"S/140°35"W, 11 Jul 1988, Florence & Teikiteetini 9410 (BISH, OS, P, PAP, PTBG, US); Main ridge, top, 400 m, 23 Mar 1960, Decker 349 (BISH, US); On top, summit plateau, 320 m, 10 Jul 1988, Perlman 10064 (BISH, F, HAW, MO, P, PAP, PTBG, UBC, US); Plateau, secteur E, 320 m, 7°55"S/140°34"W, 11 Jul 1988, Florence & Teikiteetini 9427 (BISH, CHR, P, PAP, PTBG, US); Tres commun au-dessus de 200 m, 200 m, 9 Aug 1987, Thibault 1102 (PAP).

##### Discussion.

*Bidens beckiana* is characterized by having outer and inner involucral bracts that are scarcely differentiated from the pales, and very broad outer bracts that are usually somewhat constricted at the base.

### Putative hybrid *Bidens bipontina* × *Bidens polycephala*

We have examined two gatherings from Nuku Hiva in Tapueahu Valley NW of Toovii that may represent hybrids. One of them (Butaud 2587) was collected in a small population of ca. 10 similar individuals (Butaud, pers. comm.), and could represent either hybrid individuals or less likely an undescribed taxon. A second gathering (Perlman & Wood 15021) seems to represent a mixed collection of one parent (*Bidens bipontina*, all but one of the specimens) and a putative hybrid (one specimen at US). These plants were growing with or fairly close to populations of *Bidens bipontina*, which occurs in the valley, and *Bidens polycephala*, which occurs in nearby areas, but has not been collected specifically near these putative hybrids. More information is needed to make an informed interpretation. These collections differ from both putative parents in having almost exclusively trifoliolate leaves, relatively small heads 1–3 mm in diameter excluding rays, glabrate, but with scattered hairs on margins of bracts, upper surfaces and in axils, outer involucral bracts ca. 2 mm long, linear, sparsely ciliate, ray florets 2–4 per head, rays 4–8 mm long, and achenes ca. 3 mm. The Butaud collection was included in a recent molecular phylogenetic study of Pacific *Bidens* (Funk et al. unpubl.), and it is placed in a small clade consisting of *Bidens bipontina* and *Bidens cordifolia*, but is not identical to either.

#### Specimens examined.

**Marquesas Islands. Nuku Hiva.** Ridge crest 2 valleys S of Airport road, back of Tapueahu gulch, to NW of Toovii over summit crest, 1024 m, 21 Sep 1995, Perlman & Wood 15021 (US); Vaiteheii, flanc nord de Tapueahu, 600 m, 27 Jan 2010, Butaud 2587 (PTBG).

## Supplementary Material

XML Treatment for Bidens henryi

XML Treatment for Bidens polycephala

XML Treatment for Bidens uapensis

XML Treatment for Bidens microcephala

XML Treatment for Bidens woodii

XML Treatment for Bidens evapelliana

XML Treatment for Bidens wichmanii

XML Treatment for Bidens bipontina

XML Treatment for Bidens cordifolia

XML Treatment for Bidens beckiana
